# Rhythm but not melody processing helps reading via phonological awareness and phonological memory

**DOI:** 10.1038/s41598-022-15596-7

**Published:** 2022-08-02

**Authors:** José Sousa, Marta Martins, Nathércia Torres, São Luís Castro, Susana Silva

**Affiliations:** 1 Faculty of Psychology and Educational Sciences, Center for Psychology at University of Porto, University of Porto, Porto, Portugal; 2 Instituto Universitário de Lisboa (ISCTE-IUL), Lisboa, Portugal

**Keywords:** Psychology, Human behaviour

## Abstract

Despite abundant evidence that music skills relate to enhanced reading performance, the mechanisms subtending this relation are still under discussion. The Temporal Sampling Framework (TSF) provides a well-defined explanation for the music-reading link: musical rhythm perception would relate to reading because it helps to encode speech units, which, in turn, is fundamental to reading. However, in spite of this clear mediation-based prediction (effect of music skills mediated by the encoding of speech units), the tests made to it so far remain inconclusive, either due to the use of hybrid measures (rhythm perception and production, musical and non-musical rhythm) or to underspecified mediation results (unclear presence of partial mediation). In the present study, we addressed these potential weaknesses of previous studies and investigated whether phonological memory and phonological awareness (proxies of speech encoding abilities) mediate the effects of rhythm perception abilities on reading in late first-graders. To test for the specificity of musical rhythm in this relation, we examined the same hypothesis for melody perception. Results showed full mediation for effects of musical rhythm perception, while melody perception did not even relate to reading. Our findings support the predictions embedded in the TSF and highlight the potential of rhythm-based interventions in early stimulation.

## Introduction

The relation between music skills and reading has been a topic of interest in the last decades^1–4^, but the path that leads from music to reading remains undetermined. Music and language share many characteristics and require similar processes^5–7^, but one question that remains open is *which* music-related processes—e.g., pitch (related to melody), timbre, or rhythm perception—may be important to reading, and another is *why* it might be so. Goswami^8,9^ proposed a unifying theory of dyslexia potentially extending to Developmental Language Disorder (DLD)—the Temporal Sampling Framework (TSF)—which incorporates an answer to these two questions: among music skills, *rhythm perception* would be crucial to reading, and rhythm perception would be important because it would allow effective *encoding of speech units*.

Temporal encoding is an important aspect of information encoding in the brain^10^, and temporal encoding via the synchronous activity of oscillating networks of neurons at different frequency bands (e.g., delta, 1.5–4 Hz; theta, 4–10 Hz and gamma, 30–80 Hz) is crucial in the perceptual processing of both rhythm^11^ (mostly delta and theta) and speech^12–14^ (all frequency bands). TSF postulates that rhythm perception relates to reading via effective temporal encoding of speech units at several levels: stress units (delta frequencies), syllables (theta), and phonemes (gamma). Effective encoding of speech units (at all levels) is a precondition of *phonological awareness*—the ability to reflect upon and segment the sound structure of spoken words and syllables^15,16^—which is a known predictor of reading^17^. Phonological awareness appears to develop hierarchically and, most likely, based on sensory information from the speech signal. Research results reveal that human infants show syllabic sensitivity^18^, using rhythmic cues to segment syllables and words from the acoustic signal to build a lexicon of spoken wordforms. Preliterate children and illiterate adults across languages are aware of phonological units at relatively large grain sizes (stressed syllables, syllables, and rhymes), and, as the brain learns to read in alphabetic orthographies, phonological awareness evolves into a more discrete representation of speech sounds—phoneme awareness^9^. Effective encoding of speech units is also a precondition of *phonological memory*, in that the accurate retention of speech units depends on encoding accuracy. Phonological memory is also a well-known precursor of reading^19,20^.

TSF predicts that rhythm perception is the most important music skill when it comes to predicting reading because the effective encoding of speech units (leading to effective phonological awareness and phonological memory) is supposed to depend on the frequency-tracking activity that subtends rhythm perception more than on any other type of music-related activity. This *TSF-based link* between music and reading contrasts with views that prioritize the relation between pitch perception and the encoding of speech units. For instance, it has been suggested that frequency-processing abilities, which subtend pitch perception, may be necessary to discriminate between the spectra that characterize different phonemes^21,22^. It has also been claimed that the detection of prosody-related pitch changes is important for establishing stress location and, thus, for segmenting the speech stream into units^23^. Therefore, a critical test of the TSF-based link between music and reading engages two components: (1) showing that rhythm is more important than pitch to predict reading and reading-related skills such as phonological awareness or phonological memory; and (2) demonstrating that the relation between rhythm and reading is substantiated by a *mediation* mechanism^24,25^ with phonological awareness and/or phonological memory as mediators.

Mediation occurs when the effect of an independent variable (e.g., rhythm perception) is transmitted to the dependent variable (e.g., reading) through one or more intervening variables (e.g., phonological awareness and phonological memory). It implies that the relation between independent and dependent variables diminishes or even disappears when mediators are considered. If the TSF-based link between music and reading is valid, we would expect that: (1) rhythm, more than pitch perception predicts reading (total effect); (2) rhythm predicts phonological awareness and phonological memory (3) which, in turn, predict reading (2 and 3 define an indirect effect); (4) when phonological awareness/phonological memory are incorporated as mediators in a mediation model, rhythm perception loses predictive power (the resulting direct effect of rhythm on reading decreases compared to its total effect). To our knowledge, the full critical test of the TSF-based link between music and reading has not yet been done, even though TSF-compatible results have been found, including (1) evidence of a close relation between reading and rhythmic skills, (2) evidence that rhythm perception relates to phonological awareness and that (3) phonological awareness predicts reading.

Evidence for the central role of rhythm processing in reading (1) seems reliable despite some contradictory findings. Douglas and Willatts^26^ showed that rhythm, but not pitch discrimination, was significantly related to reading ability, and several recent studies strengthened the rhythm-reading link^27–29^. However, in an older study Barwick et al.^30^ had found that melodic (pitch) discrimination was related to reading, but rhythmic discrimination was not; Anvari et al.^31^ and Moritz et al.^32^ found that rhythm discrimination was not related to reading in second-grade students; and Swaminathan et al.^33^ found no relation at all between music-perception skills and reading. The privileged relation between rhythm perception and phonological awareness (2) has been empirically supported, even though evidence is mixed. On the one hand, some studies indicate that rhythm abilities are not more closely related to phonological awareness than melodic ones: Sun et al.^34^ found that melodic, but not rhythm, discrimination relates to phonological awareness in amusic adults, and some studies pointed to the relation of both melodic and rhythm discrimination with phonological awareness^31,35^. Other studies had mixed results at different moments of assessment^1^. For example, Moritz et al.^32^ found no relation between rhythm discrimination and phonological awareness in kindergarteners, but this association became positive in the second grade. On the other hand, a considerable number of studies supports a positive relationship between rhythm discrimination and phonological awareness, thus strengthening the TSF claims at this level^16,28,29,36^. Moreover, findings on the positive association between phonological awareness and reading (3) are quite consistent^37–44^, and phonological awareness seems to be a major predictor of success in reading acquisition^17,45^.

Few studies tapped into the critical test of the TSF-based link between music and reading, which implies a (4) mediation analysis. Ozernov-Palchik et al.^16^ found that the association between musical rhythm perception and letter-sound knowledge was mediated by phonological awareness, but, as they were investigating kindergarteners, they used a precursor of reading (letter-sound knowledge) as the dependent variable. A recent study^46^ examined the hypothesis that phonological skills mediate the relation between rhythm and literacy development in third graders. Some mediation requirements were fulfilled in that both rhythm and phonological awareness predicted literacy development, but the association between rhythm skills and phonological awareness was non-significant in the mediation test. The authors suggested this might have been due to their choice of a rhythm production task, which could be less related to phonological skills than rhythm perception. Some other studies seem to speak against mediation, but conclusions remain unclear. For instance, Holliman et al.^28^ came close to the critical test by showing that rhythm processing keeps predicting reading after controlling for phonological awareness (no full mediation). However, their rhythm processing variable was hybrid because it combined speech and non-speech (musical) rhythm, as well as rhythm perception and production. In addition, their presentation of results does not clarify whether partial mediation occurred, i.e., if rhythm discrimination effects on reading diminished when phonological awareness was added to the regression. In a similar vein, Huss et al.^29^ found that rhythm discrimination still predicts reading after controlling for reading predictors (phonological awareness and working memory). Nevertheless, the effect size of the relation between rhythm discrimination and reading diminished when reading predictors were added to the regression, suggesting partial mediation.

Despite suggestions that TSF might offer a valid explanation for the relation between music skills and reading, further critical evidence is needed. On the one hand, results concerning the relative impact of rhythm vs. pitch perception on reading and phonological awareness are mixed and, according to TSF, rhythm perception should have a more prominent role. On the other hand—and critically—direct tests of the mediation mechanism implied in TSF either target non-pure variables such as rhythm production instead of perception, speech and musical rhythm instead of musical rhythm alone, reading precursors instead of reading, or show inconclusive results. To address these problems, in the present study we tested whether musical (non-speech) rhythm and melodic perception impact reading skills in children finishing their first year of school and, if so, whether the influence of rhythm perception is mediated by phonological awareness and/or phonological memory. Although the mediating role of phonological memory has not been highlighted in the literature as much as that of phonological awareness, both depend on effective encoding of speech units, and both relate to reading. For this reason, we implemented a parallel mediation model^24^, where both phonological awareness and phonological memory—the latter implemented as working memory for syllables—were incorporated as mediators.

## Results

Table 1 shows children’s results in cognitive ability, melodic and rhythmic discrimination (accuracy and *d’* scores), reading, phonological awareness, and working memory for syllables (forward and backward). Average scores in auditory discrimination were 0.62 for rhythm (SD = 0.14, range = 0.35—1.00) and 0.60 for melody (SD = 0.10, range = 0.35–0.80). Performance was above chance level (0.50) in both tasks, *p*_*s*_ < 0.001, BF_10_ > 100, and there was no substantial departure from normality (skewness, range = −0.28—0.51; kurtosis, range = −0.00–0.12)^47^. Performance level did not differ between the two tasks, *F*(1, 73) = 2.29, *p* = 0.14, *η2* = 0.03; BF_10_ = 0.51. A further analysis was conducted using *d’* scores and the results were similar, *F*(1, 73) = 0.82, *p* = 0.37, *η2* = 0.01; BF_10_ = 0.25. Additionally, we examined *C* scores in both tasks: average *C* was 0.67 (SD = 0.59) for melody and 0.17 (SD = 0.57) for rhythm. These values indicated a preference for same responses (a conservative criterion). Indeed, inspection of accuracy separately for same and different pairs revealed higher scores for same than different trials in melodic (0.81 vs. 0.38, SD = 0.21) and rhythmic (0.67 vs. 0.56, SD = 0.23) discrimination. Finally, *d'* and *c* scores differed significantly from zero in rhythm (*d’*: *t*(73) = 7.30, *p* < 0.001; *c*: *t*(73) = 2.63, *p* = 0.01) and melodic (d’: *t*(73) = 8.54, *p* < 0.001; *c*: *t*(73) = 9.74, *p* < 0.001) tasks. We used *d’* scores in further analyses.

**Table 1:** Children’s cognitive ability and performance in rhythmic and melodic discrimination, reading, and speech-related tasks.

Task	Mean (SD)	Range	Skewness	Kurtosis
RCPM (standard scores)	110.08 (16.25)	84.74–135.62	0.12	−1.41
Rhythm Discrimination (proportion of correct responses)	.62 (.14)	.35–1.00	0.51	0.12
Rhythm Discrimination (*d’* scores)	0.71 (0.84)	−0.78–3.29	−0.80	0.73
Melodic Discrimination (proportion of correct responses)	.60 (.10)	.35–.80	−0.28	−0.00
Melodic Discrimination (*d’* scores)	0.63 (0.63)	−1.03–1.90	−0.32	−0.20
Words Correct per Minute Index	26.50 (26.69)	0–91.79	0.97	−0.29
High-frequency Word Reading (items/min)	17.74 (17.68)	0–73.80	1.17	0.68
Low-frequency Word Reading (items/min)	13.69 (12.37)	0–49.80	1.04	0.43
Pseudoword Reading (items/min)	15.96 (13.07)	0–52.20	0.59	−0.60
Working Memory Syllables Forward (recalled sequences, max = 13)	2.49 (1.20)	0–5	0.34	−0.33
Working Memory Syllables Backward (recalled sequences, max = 13)	1.48 (0.80)	0–3	0.24	−0,38
Epilinguistic Phonological Awareness (correct items, max = 40)	28.18 (5.80)	17–38	−0.09	−1.11
Metalinguistic Phonological Awareness (correct items, max = 24)	10.84 (5.80)	0–24	0.43	−0.52

*N* = 74 for all analyses, except for syllable working memory (forward and backward) where *n* = 73 due to a missing value. *RCPM* Raven’s Coloured Progressive Matrices.

### Extraction of reading ability factors

Because we had four reading tasks (i.e., words correct per minute index, high- and low-frequency word reading, and pseudoword reading) that were correlated (see Supplementary Table S1), we asked whether a small set of aggregate factors could be estimated and used as indices of latent constructs. For that purpose, we conducted an exploratory factor analysis (promax rotation). Cattell’s scree plot was used to determine the adequate number of factors, and one was selected. The Kaiser–Meyer–Olkin measure of sampling adequacy was 0.87, and Bartlett’s test of sphericity was significant, *χ2*(6) = 403.44, *p* < 0.001. One factor explaining 89% of the total variance was extracted. It explained 80, 95, 93, and 89% of the variance of the words correct per minute index, high-frequency word reading, low-frequency word reading, and pseudoword reading, respectively. This factor was interpreted as *reading ability* and used in subsequent analysis.

### Sociodemographic and cognitive correlates

We examined how the main study variables—rhythm and melodic discrimination, reading ability, syllable working memory (forward and backward), and epi- and metalinguistic phonological awareness—related to age, sex, socioeconomic status, and cognitive ability (Table 2). Socioeconomic status is known to influence reading^48^. Cognitive ability is related to both reading^49^ and rhythm abilities^50^. Age influences rhythm ability^51^ and its relation with reading is trivial. None of the target variables correlated with sociodemographic and cognitive factors, with two exceptions: cognitive ability correlated with working memory for syllables (forward), *r* = 0.31, *p* = 0.01, CI 95% [0.09, 0.50], and with epilinguistic phonological awareness, *r* = 0.30, *p* = 0.01, CI 95% [0.08, 0.50]; both correlations were supported by substantial Bayesian evidence, BF_10_ = 4.76 and BF_10_ = 4.36, respectively. Thus, cognitive ability was included as a covariate of no-interest in subsequent analyses.

**Table 2:** Pearson correlations and independent sample t-tests between the main study variables—rhythm and melodic discrimination, reading ability, syllable working memory (forward and backward), and epi- and metalinguistic phonological awareness—and age, sex, socioeconomic status, and cognitive ability.

	Age (years)		Sex		Socioeconomic status		Cognitive ability	
	*r*, *p*	BF_10_	*t*, *p*	BF_10_	*t*, *p*	BF_10_	*r*, *p*	BF_10_
Rhythm Discrimination	−.05, .67	0.16	0.63, .53	0.29	0.35, .73	0.25	.06, .62	0.16
Melodic Discrimination	.03, .81	0.15	1.25, .22	0.47	0.36, .72	0.25	.04, .73	0.15
Reading Ability	−.09, .45	0.19	−.64, .52	0.29	1.44, .16	0.58	.12, .30	0.25
Working Memory Syllables Forward	.17, .14	0.42	−.93, .36	0.35	−.82, .41	0.32	**.31, .01**	4.76
Working Memory Syllables Backward	.06, .63	0.16	−.04, .97	0.24	.67, .50	0.29	.23, .05	0.91
Epilinguistic Phonological Awareness	.10, .39	0.21	−1.65, .10	0.78	.85, .40	0.33	**.30, .01**	4.36
Metalinguistic Phonological Awareness	.05, .67	0.16	−.37, .71	0.26	1.93, .06	1.18	.09, .47	0.19

*N* = 74 for all analyses, except for those involving Working Memory of Syllables, where *n* = 73 due to a missing value. Degrees of freedom (df) are equal to 72 for all independent sample t-tests, except for those involving Working Memory of Syllables, where df = 71 due to a missing value. Bold numbers indicate significant results.

### Correlations between the main variables

Correlations between rhythm discrimination, melodic discrimination, reading ability, working memory for syllables (forward and backward), and epilinguistic and metalinguistic phonological awareness are presented in Table 3 and Supplementary Fig. S1. Cognitive ability was included as a covariate of no-interest in all the correlations. Higher scores in rhythm discrimination were associated with better reading ability, working memory for syllables forward, epilinguistic phonological awareness, and metalinguistic phonological awareness. These correlations were supported by substantial evidence. No significant correlations were found between rhythm discrimination and working memory for syllables backward, but the evidence in favour of the null hypothesis was weak. We ran additional analyses testing the correlation between rhythm discrimination and each of the reading measures (i.e., words correct per minute index, high- and low-frequency word reading, and pseudoword reading). These analyses are presented in Supplementary Table S2. Melodic discrimination was not significantly correlated with reading ability, nor with working memory for syllables forward or metalinguistic phonological awareness; for these correlations, the evidence in favour of the null hypothesis was weak. It correlated positively with working memory for syllables backward, with substantial evidence supporting this association, and with epilinguistic phonological awareness, but the evidence supporting this association was weak. Reading ability was positively associated with working memory for syllables forward and backward, epilinguistic phonological awareness, and metalinguistic phonological awareness; decisive Bayesian evidence supported these relationships. Working memory and phonological awareness measures also correlated with each other, decisive Bayesian evidence supporting these correlations.

**Table 3:** Pearson correlations between rhythm discrimination, melody discrimination, reading ability, working memory for syllables (forward and backward), and epi- and metalinguistic phonological awareness, after removing the effects of cognitive ability (partial correlations).

	1	2	3	4	5	6	7
1. Rhythm Discrimination	–						
2. Melodic Discrimination	.41*** *81.24*	–					
3. Reading Ability	.30** *4.11*	.19 *0.53*	–				
4. Working Memory Syllables Forward	.31** *5.24*	.23 0.91	.58*** > *100*	–			
5. Working Memory Syllables Backward	.21 *0.72*	.31** *5.08*	.47*** > *100*	.36** *15.65*	–		
6. Epilinguistic Phonological Awareness	.33** *7.91*	.25* *1.39*	.55*** > *100*	.49*** > *100*	.49*** > *100*	–	
7. Metalinguistic Phonological Awareness	.33** *7.76*	.20 *0.60*	.69*** > *100*	.47*** > *100*	.45*** > *100*	.56*** > *100*	–

*N* = 74 for all analyses, except for those involving Working Memory of Syllables, where *n* = 73 due to a missing value. BF_10_ values are indicated in italics. *p < .05; **p < .01; ***p < .001.

Given the lack of correlation between melodic discrimination (predictor) and reading ability (outcome), we did not move on with mediation tests for melodic discrimination. Rhythm discrimination (predictor) fulfilled the requirement of correlating with reading ability (outcome). It also correlated with three out of four mediators, namely working memory for syllables forward, epilinguistic phonological awareness, and metalinguistic phonological awareness (not with working memory for syllables backward). Therefore, we moved on with a mediation model that included rhythm discrimination as a predictor, three mediators, and reading ability as outcome.

### Mediation tests

**Figure 1:**
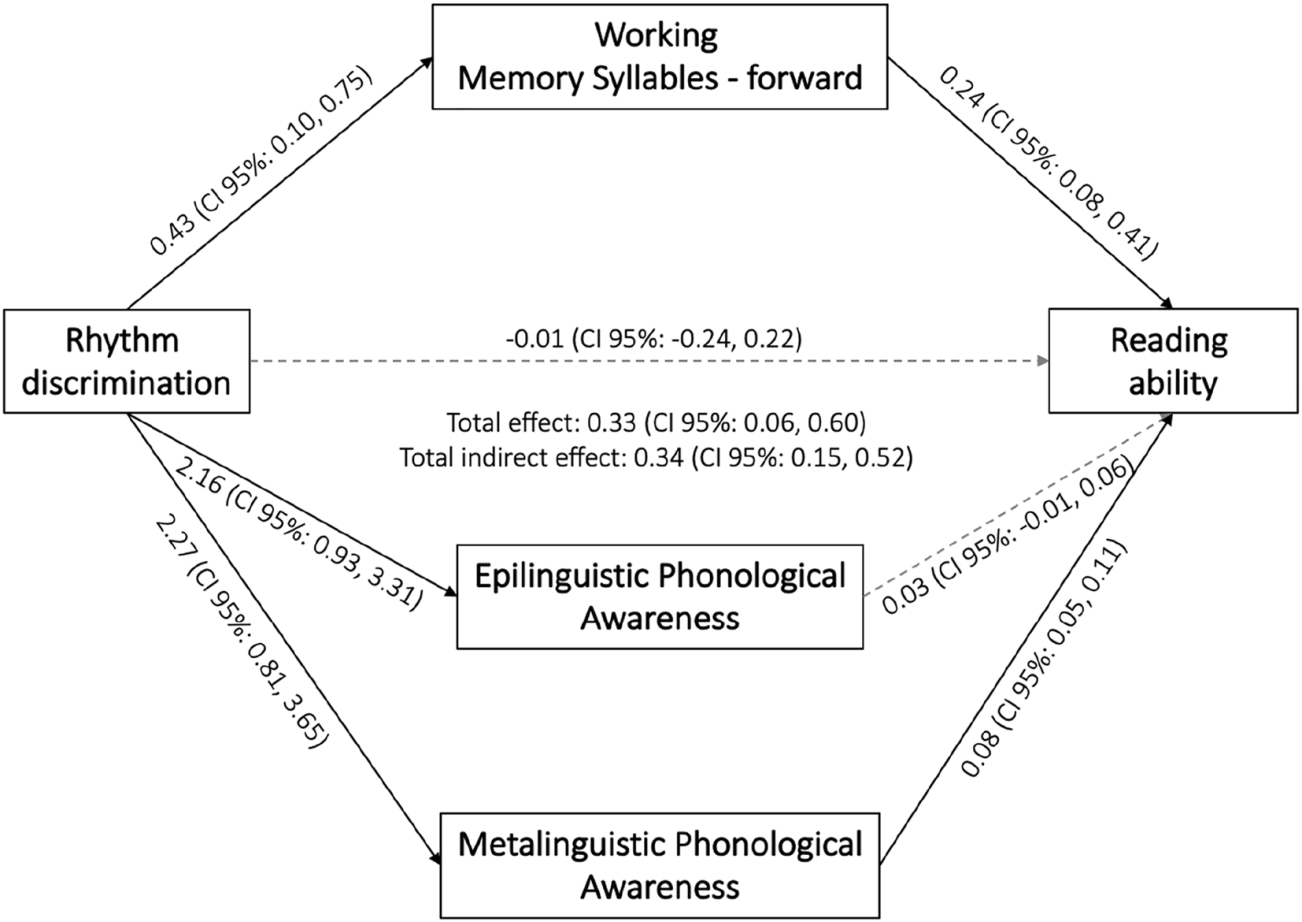
Parallel mediation model (N = 73) depicting the mediation effect of working memory of syllables forward, epilinguistic phonological awareness, and metalinguistic phonological awareness on the association between rhythm discrimination and reading ability. The model was controlled for cognitive ability. Effects were considered significant when the CIs did not include 0. Inference was based on percentile bootstrap 95% confidence intervals (CIs) with 20,000 samples.

To evaluate whether and to which extent the association between rhythm discrimination and reading ability was mediated by working memory for syllables forward, epilinguistic phonological awareness, and metalinguistic phonological awareness, we conducted a parallel mediation model. Results based on 20,000 bootstrapped samples indicated that, controlling for cognitive ability, the total effect of rhythm discrimination on reading ability was significant (0.33, 95% CI: LL = 0.06 to UL = 0.60) but the direct effect was not (-0.01, 95% CI: LL = −0.24 to UL = 0.22), and indirect effects were present (Fig. 1). Overall, the three mediators fully mediated the relationship between rhythm discrimination and reading ability (Indirect effect _overall_ = 0.34, 95% CI: LL = 0.15 to UL = 0.52) indicating that children with better rhythm discrimination were more likely to have higher performance on syllable working memory forward, epilinguistic and metalinguistic phonological awareness. Children with better performance on syllable working memory forward and on epilinguistic and metalinguistic phonological awareness were also more likely to have better reading ability. Two of these three mediators contributed significantly to the overall indirect effect: working memory for syllables forward (0.11, 95% CI: LL = 0.01 to UL = 0.23) and metalinguistic phonological awareness (0.18, 95% CI: LL = 0.06 to UL = 0.32). Epilinguistic phonological awareness did not mediate the relationship between rhythm discrimination and reading ability (0.06, 95% CI: LL = −0.03 to UL = 0.13). Indirect effects of syllable working memory forward and metalinguistic phonological awareness did not differ significantly (−0.08, 95% CI: LL = −0.24 to UL = 0.09). The effect size resulting from running this model was *f*^2^ = 1.38, which is above the minimal detectable effect for our sample (i.e., *f*^2^ = 0.18 for four predictors).

For completeness, we repeated the above correlations and mediation analyses without controlling for cognitive ability, and the results showed a similar pattern (see Supplementary Table S3 and S4, and Supplementary Fig. S2).

## Discussion

Our goal was to test the predictions embedded in the Temporal Sampling Framework regarding the relation between music and reading skills. We defined a TSF-based link between music and reading, according to which rhythm, but not pitch, perception relates to reading, and the relation between rhythm perception and reading would be mediated by phonological awareness and/or phonological memory. Support to the TSF-based link would be revealed by the co-presence of a specific set of associations: first, musical rhythm perception—more than melodic perception—relating to reading skills and to the outcomes of effective encoding of speech units (phonological awareness and/or phonological memory); second, phonological awareness and/or phonological memory relating to reading; final, and critically, a mediation test should show that the relation between rhythm perception and reading weakens or even vanishes when phonological awareness and/or phonological memory are taken as a mediators. While previous studies included approximations to a mediation test of this sort, they have either used non-pure measures of rhythm^28,46^ and reading^16^, or have presented unclear results regarding the presence of mediation^29^. To address these gaps, we focused on the purest implementations of TSF relevant variables—rhythm perception, excluding production, and reading ability, excluding reading precursors—and quantified mediation through dedicated mediation tests. In addition, we implemented the concept of speech units encoding in a broad, multifaceted approach, including not only the variable phonological awareness, which has dominated research on this matter, but also memory for speech units (working memory for syllables).

Our results have supported the TSF-based predictions. (1) Rhythm, but not melody, perception correlated with reading; (2) rhythm perception related to phonological awareness (epilinguistic and metalinguistic) and syllable working memory forward, while melody perception only related to syllable working memory backward; (3) phonological awareness and syllable working memory related to reading; (4) rhythm perception ceased to predict reading when phonological awareness and syllable working memory forward were entered as mediators. Mediation results indicate that, as proposed by TSF, rhythm perception as such is not key to reading. Instead, what seems to be key to reading is the cognitive component of rhythm perception that is common to the effective encoding of speech units.

In the context of the current literature, our findings make additional contributions. They suggest that letter-to-sound knowledge may be a proxy to reading, since Ozernov-Palchik et al.^16^ used letter-to-sound knowledge instead of reading proper and also found evidence for mediation. In a different perspective, our results also suggest that previous studies showing no evidence of mediation may have failed to do so either because they included rhythm production^46^—which engages processes other than the ones akin to frequency-tracking, such as motor processes—or combined musical (regular) rhythm with speech (irregular) rhythm^28^. Finally, the relevance of phonological memory as a mediator between rhythm perception and reading—untested so far, to our knowledge—is consistent with the idea that the efficient encoding of speech units, allowing not only phonological awareness, but also phonological memory, is a key foundation of reading. Nevertheless, we should keep in mind that we did not measure encoding in a direct way, and we are just assuming that phonological awareness and phonological memory reflect encoding.

Although generally consistent with the TSF-based link between music and reading, the mediation model showed two deviations from our hypothesis. First, working memory backward (one component of phonological memory) did not correlate with rhythm discrimination, unlike working memory forward (the other component). One explanation for this might be that working memory backward engages levels of cognitive processing that go beyond the requirements of rhythm discrimination, in the sense of engaging not only maintenance in memory (common to rhythm processing), but also mental manipulation (not obvious in rhythm processing). Interestingly, working memory backward correlated with melodic discrimination. This might indicate that mental manipulation may be more important to melodic than rhythmic processing, and would be consistent with the continuous comparisons between pitch values required by relative pitch processing (unlike rhythm, where a single beat reference may be enough). Second, epilinguistic phonological awareness, though related with both rhythm perception and reading, was not a significant mediator, contrasting with metalinguistic phonological awareness. We have no obvious explanation for this, but it is worth mentioning that we ran separate mediation models before running the parallel model, and epilinguistic phonological awareness was, then, a significant mediator (please see Supplementary Fig. S3). Given that parallel mediation models were designed to accommodate correlations between different mediators^24^, and correlations between the two types of phonological awareness were strong, it is likely that the null contribution of epilinguistic phonological awareness as a mediator in the final model reflects statistical compensations inherent to parallel mediation models.

Among the limitations of our study, we highlight the ambiguities that remain on causality issues: even though we are testing a causal path with a statistical tool that stands on causal assumptions (mediation), the cross-sectional design we used does not allow definite causal statements. For that, we would need longitudinal results. Nevertheless, we should not forget that the relation between rhythm perception and reading is not quite bidirectional, i.e., rhythm perception is less dependent on experience (instruction) than reading and, in this sense, causality from rhythm to reading is more likely than causality from reading to rhythm. Another limitation is that causality relations between phonological memory/phonological awareness and reading are likely bidirectional, and we tested for one direction only. In a similar vein, it is likely that working memory for syllables influences phonological awareness, even though it has been suggested that phonological memory (particularly serial order memory) has an independent influence on reading development, over and above phonological awareness^20^. We chose a limited set of causality paths driven by theory, but we do not exclude the possibility that more complex models may provide a more accurate picture of the relations between rhythm, reading precursors and reading. Future studies could address this.

The idea that synchronizing brain oscillations to musical rhythm is equivalent to synchronizing brain oscillations to speech units (the reason why musical rhythm perception relates to reading) is disputable: unlike musical rhythm, the rhythm of speech is not isochronous—stress units, syllables, and phonemes have variable durations. The anisochrony of speech is more evident in stress-timed languages, in which syllable length varies substantially due to vocalic reduction, than in syllable-timed languages like French. European Portuguese combines syllable-timing with stress-timing, and thus it is substantially exposed to anisochrony^52^. So, the following questions emerge. Could it be that isochrony is irrelevant, and either the brain synchronizes its oscillations in a flexible manner such that they adjust to speech irregularities, or regular brain oscillations can “live with” the irregularities of speech? Or is it rather that—as proposed by Ozernov-Palchik and Patel^6^—as a result of accurately processing musical rhythm (an ability that engages prediction) one becomes a skilled predictor and this, in turn, helps sequential processing in speech regardless of timing? Or maybe there is another explanation for the imperfect partnership between feeling the beat and sensing speech units. Future research could shed light on this.

Regarding the role of language-specific rhythm characteristics, it is important to note that an overgeneralization of our findings—as any findings from a particular language—is not recommended. Even though we were dealing with some anisochrony in speech, we worked with a language, European Portuguese, of an intermediate rhythmic class that combines stressed-timed (e.g., vowel reduction) with syllable-timed characteristics (e.g., open syllables). We may, thus, wonder whether the same pattern would emerge as isochrony decreases. In general, it would be wise to interpret findings on the relation between rhythm, pitch and reading considering the characteristics of the participants’ native language. Pitch plays a prominent role in tonal languages, where it changes the meaning of a word, but it may also be relevant in non-tonal languages where the placement of pitch accents may change a word’s meaning^53^.

Other follow-ups to our study could address developmental issues. We found evidence that phonological awareness and phonological memory mediate the relation between musical rhythm and reading in beginning readers. Ozernov-Palchik et al.^16^ found a similar result with kindergarteners, though replacing reading with letter-to-sound naming. However, null relations between rhythm and reading have been found for second graders^31,32^. Given that phonological awareness predicts reading less and less as schooling advances^38,54,55^, should we expect that the mediation-based mechanism loses power throughout the school years? Also, given that the most salient units of music rhythm tend to lie within delta and theta ranges (1000 ms to 100 ms wavelengths) corresponding to stress and syllabic units, what happens when phonological awareness refines into the phoneme level and starts focusing on the gamma band (33–12 ms wavelengths)—would musical rhythm still correlate with reading? Furthermore, what happens in older children (around 9–10 years old), once they have achieved a certain level of proficiency, and reading relies more and more on the lexical route? Research suggests that, at this stage, Rapid Automatized Naming (RAN) becomes a better predictor of reading than phonological awareness^56–59^. Does this weaken the relation between musical rhythm and reading?

These and many other exciting questions remain open. The practical suggestion of our study, together with that of Ozernov-Palchik et al.^16^, is that accurate perception of musical rhythm may foster early reading abilities via phonological memory and phonological awareness. In this sense, rhythm training programs—be they part of music classes or stand-alone programs—may be an important tool in early stimulation. Nevertheless, before this can be implemented based on solid grounds, longitudinal studies are needed to test the mediation path we propose here.

## Methods

### Participants

Seventy-four children participated in this study (mean age = 7.33 years, *SD* = 0.35, range = 5.87–7.88, 32 boys). They were all monolingual native speakers of European Portuguese and attended the first grade at public schools from the Porto area (Northern Portugal). None of the children had known hearing, visual, intellectual, or behavioural disorders. Regarding parental education, most of their parents had completed the equivalent to middle school (mothers: 46%, fathers: 45%), some attended high school (mothers: 24%; fathers: 19%), and others higher education (mothers: 22%; fathers: 16%). A few attended only elementary school (mothers: 7%; fathers: 9%) or did not give any information about their school level (mothers: 1%; fathers: 11%). The socioeconomic status of the participants was evaluated based on information regarding support received from the Portuguese social security system. In Portugal, school-aged children get financial aid for lunch, school material and field trips according to their household income, making such an aid a proxy for socioeconomic status. In our sample, 47% of the children benefited from this support, while 53% got none. Children were tested as part of a wider project looking at music training and auditory processing in primary education. Written informed consent was obtained from children’s parents or legal guardians, and children gave their verbal assent before data collection started. The study was approved by the ethics committee of the Faculty of Psychology and Educational Sciences at University of Porto (FPCEUP 2015.1.23) and the school boards, and it was conducted in agreement with the Declaration of Helsinki and later amendments.

### Measures

*Raven’s Coloured Progressive Matrices*^60^. The Raven’s Coloured Progressive Matrices (RCPM) test measures general non-verbal cognitive ability. It is composed of three series of 12 items of increasing complexity. The maximum score is 36 points. Raw scores were converted to standard scores (*M* = 100, *SD* = 15) based on Simões’s age norms for Portuguese children^61^.

*Melodic and Rhythm Discrimination*^62^. Melodic and rhythm discrimination tasks are part of the Music Aptitude Tests that were developed by Overy and colleagues^62^ and later revised by Moore^63^. We used this revised version. Each task takes 10–15 min and consists of two practice and 20 test trials. Trials comprise a target melody/rhythm and a comparison sequence, whose similarity children are asked to evaluate using a forced-choice procedure (i.e., same-different paradigm). In each task, 10 test trials are identical and 10 different. Stimuli are presented at a tempo of 100 beats per minute and increasing level of difficulty. Each trial is rated as correct or incorrect for a total of 20 points per task. In the Melodic Discrimination test, each same/different item is composed of two tonal melodies separated by a 3-s pause. The rhythm is isochronous, meaning that all sounds have the same duration. In different items, a single note marks the difference between the two melodies (melodic change). The test starts with three-note melodies and melodic differences of three semitones. The length of the melodies increases from three to six notes and melodic changes diminish to one semitone as the difficulty increases, while preserving the melodic up-down contour within each pair. The Rhythm Discrimination test comprises same/different items with two rhythms also separated by a 3-s pause. It begins with short rhythms using no more than two different durations (crotchets, quavers). The level of difficulty increases as rhythms get longer and include a larger variety of durations (crotchets, quavers, dotted rhythms, triplets and rests). The rhythmic changes introduced in different pairs consist of substituting the rhythm of one beat by another rhythm with the same length (e.g., crotchet replaced by two quavers). The raw score is the proportion of correct trials over total trials. We also calculated *d′* scores (a sensitivity index) for each participant.

*Working Memory for Syllables (3DM)*^64,65^. In the syllable working memory subtest from the Differential Diagnosis of Dyslexia Maastricht battery (3DM)^64,65^, the task is to repeat, in forward and backward order, sequences of syllables presented auditorily. One point is given for each correctly repeated sequence (max = 13 points for each order).

*Epilinguistic and Metalinguistic Phonological Awareness (ALEPE)*^66^. Phonological awareness was tested for two linguistic units—syllable and rime (part-of-syllable, see below). Two different tasks of ALEPE (European Portuguese Reading Assessment Battery)^66^ were used: a same-different task (Epilinguistic Phonological Awareness–EPA) and a common unit task (Metalinguistic Phonological Awareness–MPA). In the same-different task, the child has to judge whether a pair of words share a segment (e.g., for the syllable pairs ***ru***de—***ru***mo → “Same”, ***pal***co—***for***ça → “Different”; for the rime pairs b***ol***so—p***ol***pa → “Same”, s***a***la—t***in***to → “Different”). In the common unit task, the child is asked to say aloud the shared segment (e.g., for the syllable pair ***po***vo—***po***ça → “**po**”; for the rime pair f***u***ga—l***u***me → “**u**”). One point is awarded for each correct answer. Syllable and rime scores were added in both subtests, up to a maximum of 40 points for EPA and 24 points for MPA.

*Reading*. Reading ability was assessed with the word and pseudoword reading subtests from the Differential Diagnosis of Dyslexia Maastricht battery (3DM)^64,65^, and the Words Correct per Minute (WCPM) test^67^. The 3DM subtests provide measures of high- and low-frequency word reading and pseudoword reading. Words/pseudowords are presented in columns, and the participant reads aloud as many items as possible within 30 s (maximum score per subtest: 75 items). The raw score is the number of correctly read words/pseudowords in 30 s, which is then converted into rate per minute. The WCPM test consisted of a text from a children’s tale (“O Tigre Que Veio Tomar Chá”, “The Tiger Who Came for Tea”^68^) that the children were asked to read as quickly and accurately as possible within a time limit of 1 min. The score was the number of words correctly read per minute.

### Procedure

Before data collection, parents completed a questionnaire that asked for demographic information and the child’s history of health issues. Information regarding support received from the social security system was also collected, that is, whether the child had the right to free or price-reduced school meals (with support) or if no such reduction was applicable (no support). Children were tested individually by two certified psychologists in the last trimester of the school year. Testing sessions took place in a quiet room at the children's school.

### Data analysis

Signal detection theory was applied to measure behavioural sensitivity (*d’*) and response bias (*c*) in rhythm and melodic discrimination tasks. Extreme values of hits and false alarms (i.e., 0 and 1) were replaced with 0.5/*n* and (*n−*0.5)/*n*, respectively, where *n* is the number of target or distractor trials^69^; *d’* and *c* scores were calculated as follows: *d’* = *z(hit rate)*−*z(false alarm rate)*, *c* = − [*z(hit rate)* + *z(false alarm rate)*]/2. Data were statistically analysed using standard frequentist and Bayesian approaches^70^. For each analysis, a Bayes Factor (BF_10_) was estimated; this factor considers the likelihood of the observed data given the alternative and null hypotheses. Analyses were computed on JASP Version 0.14.1 (JASP Team, 2020) using default priors. BF_10_ values were interpreted following Jeffreys’ guidelines^70,71^, that is, values between 1—3 indicate weak/anecdotal evidence for the alternative hypothesis, between 3—10 substantial evidence, between 10—30 strong evidence, between 30—100 very strong evidence, and over 100 decisive evidence. BF_10_ values below 1 correspond to evidence in favour of the null hypothesis: values below 0.33 to substantial evidence and below 0.10 to strong evidence.

We used Pearson correlations to inspect the association between the diverse study variables and, thus, evaluate mediation requirements. Testing mediation requires that the independent variable (rhythm perception, melody perception) correlates significantly with the dependent variable (reading), as well as with the mediator (phonological awareness and phonological memory)^25^. Frequentist and Bayesian approaches were combined to better inform our inferences on the tested relationships. While *p*-values only allow to accept/reject the null hypothesis, the Bayes Factor informs whether evidence supports (or not) the null/alternative hypotheses. Mediation analysis was computed using the PROCESS macro for SPSS (Version 3.5.3)^72^, with statistical inferences based on percentile bootstrap 95% confidence intervals (CIs) with 20,000 samples. Total, direct, and indirect (mediated) effects were estimated and considered significant when the CIs did not include 0. As in this study we worked with an existing dataset, we conducted a sensitivity power analysis to determine which effect size our sample size would be sensitive enough to detect. We used G*Power (version 3.1.9.4)^73^ to conduct the sensitivity power analysis; the statistical test method used the *F*-test for multiple linear regression: fixed model, R^2^ deviation from zero. The analysis with ɑ = 0.05 and power = 0.80 indicated that our sample was sufficiently large to detect a small to medium effect (*f*^2^ = 0.14 for two predictors to *f*^2^ = 0.19 for five predictors).

## Supplementary Information

Supplementary Information.

## Data Availability

The full data set can be found here: https://osf.io/hkdtn/?view_only=e79a126fc3e042cca82025194b6005a9.

## References

[1] Forgeard M (2008). The relation between music and phonological processing in normal-reading children and children with dyslexia. Music Percept..

[2] Hallam S (2019). Can a rhythmic intervention support reading development in poor readers?. Psychol. Music.

[3] Patscheke H, Degé F, Schwarzer G (2019). The effects of training in rhythm and pitch on phonological awareness in four- to six-year-old children. Psychol. Music.

[4] Thomson JM, Goswami U (2008). Rhythmic processing in children with developmental dyslexia: Auditory and motor rhythms link to reading and spelling. J. Physiol. Paris.

[5] Atherton RP (2018). Shared processing of language and music: evidence from a cross-modal interference paradigm. Exp. Psychol..

[6] Ozernov-Palchik O, Patel AD (2018). Musical rhythm and reading development: Does beat processing matter?: Rhythm and reading development. Ann. N.Y Acad. Sci..

[7] Yu M (2017). The shared neural basis of music and language. Neurosci..

[8] Goswami U (2011). A temporal sampling framework for developmental dyslexia. Trends Cogn. Sci..

[9] Goswami U (2018). A neural basis for phonological awareness? An oscillatory temporal-sampling perspective. Curr. Dir. Psychol. Sci..

[10] Buzsáki G, Draguhn A (2004). Neuronal oscillations in cortical networks. Science.

[11] Large EW, Herrera JA, Velasco MJ (2015). Neural networks for beat perception in musical rhythm. Front. Syst. Neurosci..

[12] Ding N, Melloni L, Zhang H, Tian X, Poeppel D (2016). Cortical tracking of hierarchical linguistic structures in connected speech. Nat. Neurosci..

[13] Gross J (2013). Speech rhythms and multiplexed oscillatory sensory coding in the human brain. PLoS Biol..

[14] Obleser J, Kayser C (2019). Neural entrainment and attentional selection in the listening brain. Trends Cogn. Sci..

[15] Knoop-van Campen CAN, Segers E, Verhoeven L (2018). How phonological awareness mediates the relation between working memory and word reading efficiency in children with dyslexia. Dyslexia.

[16] Ozernov-Palchik O, Wolf M, Patel AD (2018). Relationships between early literacy and nonlinguistic rhythmic processes in kindergarteners. J. Exp. Child Psychol..

[17] Tibi S, Kirby JR (2018). Investigating phonological awareness and naming speed as predictors of reading in Arabic. Sci. Stud. Read..

[18] Mehler J, Jusczyk P, Lambertz G, Halsted N, Bertoncini J, Amiel-Tison C (1988). A precursor of language acquisition in young infants. Cognition.

[19] Cunningham AJ, Burgess AP, Witton C, Talcott JB, Shapiro LR (2021). Dynamic relationships between phonological memory and reading: A five year longitudinal study from age 4 to 9. Dev. Sci..

[20] Martinez Perez T, Majerus S, Poncelet M (2012). The contribution of short-term memory for serial order to early reading acquisition: Evidence from a longitudinal study. J. Exp. Child Psychol..

[21] Bohn OS, Polka L (2001). Target spectral, dynamic spectral, and duration cues in infant perception of German vowels. J. Acoust. Soc. Am..

[22] Steinbrink C, Klatte M, Lachmann T (2014). Phonological, temporal and spectral processing in vowel length discrimination is impaired in German primary school children with developmental dyslexia. Res. Dev. Disabil..

[23] Ziegler JC, Pech-Georgel C, George F, Foxton JM (2012). Global and local pitch perception in children with developmental dyslexia. Brain Lang..

[24] Kane L, Ashbaugh AR (2017). Simple and parallel mediation: A tutorial exploring anxiety sensitivity, sensation seeking, and gender. Quant. Meth. Psych..

[25] MacKinnon DP, Fairchild AJ, Fritz MS (2007). Mediation analysis. Annu. Rev. Psychol..

[26] Douglas S, Willatts P (1994). The relationship between musical ability and literacy skills. J. Res. Read..

[27] Flaugnacco E (2014). Rhythm perception and production predict reading abilities in developmental dyslexia. Front. Hum. Neurosci..

[28] Holliman AJ, Wood C, Sheehy K (2010). The contribution of sensitivity to speech rhythm and non-speech rhythm to early reading development. Educ. Psychol..

[29] Huss M, Verney JP, Fosker T, Mead N, Goswami U (2011). Music, rhythm, rise time perception and developmental dyslexia: Perception of musical meter predicts reading and phonology. Cortex.

[30] Barwick J, Valentine E, West R, Wilding J (1989). Relations between reading and musical abilities. Br. J. Educ. Psychol..

[31] Anvari SH, Trainor LJ, Woodside J, Levy BA (2002). Relations among musical skills, phonological processing, and early reading ability in preschool children. J. Exp. Child Psychol..

[32] Moritz C, Yampolsky S, Papadelis G, Thomson J, Wolf M (2013). Links between early rhythm skills, musical training, and phonological awareness. Read. Writ..

[33] Swaminathan S, Schellenberg EG, Venkatesan K (2018). Explaining the association between music training and reading in adults. J. Exp. Psychol. Learn..

[34] Sun Y, Lu X, Ho HT, Thompson WF (2017). Pitch discrimination associated with phonological awareness: Evidence from congenital amusia. Sci. Rep..

[35] Degé F, Kubicek C, Schwarzer G (2015). Associations between musical abilities and precursors of reading in preschool aged children. Front. Psychol..

[36] Gordon RL (2015). Musical rhythm discrimination explains individual differences in grammar skills in children. Dev. Sci..

[37] Hulme C (2002). Phoneme awareness is a better predictor of early reading skill than onset-rime awareness. J. Exp. Child Psychol..

[38] Kirby JR, Parrila RK, Pfeiffer SL (2003). Naming speed and phonological awareness as predictors of reading development. J. Educ. Psychol..

[39] McBride-Chang C, Kail RV (2002). Cross-cultural similarities in the predictors of reading acquisition. Child Dev..

[40] Melby-Lervåg M, Lyster S-AH, Hulme C (2012). Phonological skills and their role in learning to read: A meta-analytic review. Psychol. Bull..

[41] Muter V, Hulme C, Snowling MJ, Stevenson J (2004). Phonemes, rimes, vocabulary, and grammatical skills as foundations of early reading development: Evidence from a longitudinal study. Dev. Psychol..

[42] Sucena A, Castro SL, Seymour P (2009). Developmental dyslexia in an orthography of intermediate depth: the case of European Portuguese. Read. Writ..

[43] Vale APS, Sousa J (2017). Tipo de erros e dificuldades na escrita de palavras de crianças portuguesas com dislexia [Type of errors and difficulties in writing words in Portuguese children with dyslexia]. Da Investigação às Práticas: Estudos de Natureza Educacional.

[44] Ziegler JC (2010). Orthographic depth and its impact on universal predictors of reading: A cross-language investigation. Psychol. Sci..

[45] Diamanti V (2017). Preschool phonological and morphological awareness as longitudinal predictors of early reading and spelling development in Greek. Front. Psychol..

[46] Lê M (2020). Rhythm in the blood: The influence of rhythm skills on literacy development in third graders. J. Exp. Child Psychol..

[47] Curran PJ, West SG, Finch JF (1996). The robustness of test statistics to nonnormality and specification error in confirmatory factor analysis. Psychol. Methods.

[48] Dolean D, Melby-Lervåg M, Tincas I, Damsa C, Lervåg A (2019). Achievement gap: Socioeconomic status affects reading development beyond language and cognition in children facing poverty. Learn. Instr..

[49] Peng P, Wang T, Wang C, Lin X (2019). A meta-analysis on the relation between fluid intelligence and reading/mathematics: Effects of tasks, age, and social economics status. Psychol. Bull..

[50] Mosing MA, Verweij KJH, Madison G, Ullén F (2016). The genetic architecture of correlations between perceptual timing, motor timing, and intelligence. Intelligence.

[51] Mastrokalou N, Hatziharistos D (2007). Rhythmic ability in children and the effects of age, sex, and tempo. Percept. Mot. kills.

[52] Seymour PHK, Aro M, Erskine JM (2003). Foundation literacy acquisition in European orthographies. Br. J. Psychol..

[53] Li X, Yang Y, Hagoort P (2008). Pitch accent and lexical tone processing in Chinese discourse comprehension: An ERP study. Brain Res..

[54] Hogan TP, Catts HW, Little TD (2005). The relationship between phonological awareness and reading: Implications for the assessment of phonological awareness. Lang. Speech Hear Serv. Sch..

[55] Leppanen U, Nieme P, Aunola K, Nurmi JE (2006). Development of reading and spelling Finnish from preschool to grade 1 and grade 2. Sci. Stud. Read.

[56] Cohen M, Mahé G, Laganaro M, Zesiger P (2018). Does the relation between rapid automatized naming and reading depend on age or on reading level? A behavioral and ERP study. Front. Hum. Neurosci..

[57] De Jong PF (2011). What discrete and serial rapid automatized naming can reveal about reading. Sci. Stud. Read..

[58] Parrila R, Kirby JR, McQuarrie L (2004). Articulation rate, naming speed, verbal short-term memory, and phonological awareness: Longitudinal predictors of early reading development?. Sci. Stud. Read..

[59] Van Den Bos KP, Zijlstra BJH, Lutje Spelberg HC (2002). Life-span data on continuous-naming speeds of numbers, letters, colors, and pictured objects, and word-reading speed. Sci. Stud. Read..

[60] Raven JC (1947). Coloured progressive matrices sets A.

[61] Simões, M. R. O Teste das Matrizes Progressivas Coloridas de Raven [The Raven Coloured Progressive Matrices test (RCPM)] in *Provas Psicológicas em Portugal [Psychological tests in Portugal]* (eds. Almeida, L., Simões, M. & Gonçalves, M.) 1–18 (Associação dos Psicólogos Portugueses, 1995).

[62] Overy K, Nicolson RI, Fawcett AJ, Clarke EF (2003). Dyslexia and music: Measuring musical timing skills. Dyslexia.

[63] Moore, E. *Music and dyslexia: Investigating the role of auditory-motor timing skills in the transfer from musical learning to language skills*. PhD dissertation (University of Edinburgh, 2018).

[64] Blomert, L. & Vaessen, A. *3DM differential diagnostics for dyslexia, cognitive analysis of reading and spelling* (Boom Test Publishers, 2009).

[65] Pacheco, A. *Caraterização cognitiva de perfis de leitores: o estudo da ortografia portuguesa* [Readers’ cognitive profiling: a study of Portuguese orthography]. Ph.D. dissertation at https://sapientia.ualg.pt/bitstream/10400.1/6076/1/finalMM.pdf (2012).

[66] Sucena, A. & Castro, S. L. *ALEPE: Bateria de provas de avaliação da leitura em Português Europeu [Test battery for reading assessment in European Portuguese.]* (CECOC, 2011).

[67] Fuchs LS, Fuchs D, Hosp MK, Jenkins JR (2001). Oral reading fluency as an indicator of reading competence: A theoretical, empirical, and historical analysis. Sci. Stud. Read..

[68] Kerr, J. *O tigre que veio tomar chá [The Tiger Who Came for Tea]* (Kalandraka, 2010).

[69] Macmillan NA, Kaplan HL (1985). Detection theory analysis of group data: Estimating sensitivity from average hit and false-alarm rates. Psychol. Bull..

[70] Jarosz AF, Wiley J (2014). What are the odds? A practical guide to computing and reporting Bayes factors. J. Probl. Solving.

[71] Jeffreys H (1961). Theory of probability.

[72] Hayes AF (2018). Introduction to mediation, moderation, and conditional process analysis: A regression-based approach.

[73] Faul F, Erdfelder E, Lang AG, Buchner A (2007). G*Power 3: A flexible statistical power analysis program for the social, behavioral, and biomedical sciences. Behav. Res. Methods.

