# Supplementary material for: Rhythm but not melody processing helps reading via phonological awareness and phonological memory
Source: Sci Rep. 2022 Aug 2;12:13224. doi: 10.1038/s41598-022-15596-7 (PMC9346111; doi:10.1038/s41598-022-15596-7)
Supplement: Supplementary file 1 — Supplementary Information. [file 41598_2022_15596_MOESM1_ESM.docx]

**Supplementary Information to Rhythm but not melody processing halps reading via phonologicalawareness and phonological memory**

**Supplementary Table S1.** Pearson correlations between reading measures (*N* = 74).

|  | 1. | 2. | 3. | 4. |
| --- | --- | --- | --- | --- |
| 1. Words Correct per Minute Index | - |  |  |  |
| 2. High-frequency Word Reading | .87***  *> 100* | - |  |  |
| 3. Low-frequency Word Reading | .85***  *> 100* | .94***  *> 100* | - |  |
| 4. Pseudoword Reading | .86***  *> 100* | .92***  *> 100* | .91***  *> 100* | - |
| *Note*. BF_10_ values are indicated in italics. *** *p* < .001. | | | | |

**Supplementary Table S2**. Pearson correlations between rhythm discrimination and Words Correct per Minute index, high- and low-frequency word reading, and pseudoword reading, after removing the effects of cognitive ability (partial correlations; *N* = 74).

|  | Rhythm Discrimination | |
| --- | --- | --- |
|  | *r*, *p* | BF_10_ |
| Words Correct per Minute Index | .31, .01 | 4.94 |
| High-frequency Word Reading (items/min) | .31, .01 | 4.42 |
| Low-frequency Word Reading (items/min) | .29, .01 | 2.87 |
| Pseudoword Reading (items/min) | .27, .02 | 1.88 |

**Supplementary Table S3**. Pearson correlations between rhythm discrimination, melodic discrimination, reading ability, working memory for syllables (forward and backward), and epilinguistic and metalinguistic phonological awareness.

|  | 1. | 2. | 3. | 4. | 5. | 6. | 7. |
| --- | --- | --- | --- | --- | --- | --- | --- |
| 1. Rhythm Discrimination | - |  |  |  |  |  |  |
| 2. Melodic Discrimination | .41***  *85.06* | - |  |  |  |  |  |
| 3. Reading Ability | .31**  *4.56* | .19  *0.55* | - |  |  |  |  |
| 4. Working Memory Syllables Forward | .33**  *6.39* | .23*  0.98 | .59***  *> 100* | - |  |  |  |
| 5. Working Memory Syllables Backward | .22  *0.87* | .32**  *5.51* | .48***  *> 100* | .40***  *59.73* | - |  |  |
| 6. Epilinguistic Phonological Awareness | .33**  *8.29* | .25*  *1.40* | .56***  *> 100* | .54***  *> 100* | .53***  *> 100* | - |  |
| 7. Metalinguistic Phonological Awareness | .33**  *8.45* | .20  *0.62* | .69***  *> 100* | .47***  *> 100* | .46***  *> 100* | .56***  *> 100* | - |
| *Note*. *N* = 74 for all analyses, except for those involving Working Memory of Syllables, where *n* = 73 due to a missing value. BF_10_ values are indicated in italics. * *p* < .05; ** *p* < .01; *** *p* < .001. | | | | | | | |

**Supplementary Table S4**. Pearson correlations between rhythm discrimination and Words Correct per Minute index, high- and low-frequency word reading, and pseudoword reading (*N* = 74).

|  | Rhythm Discrimination | |
| --- | --- | --- |
|  | *r*, *p* | BF_10_ |
| Words Correct per Minute Index | .31, .01 | 5.42 |
| High-frequency Word Reading (items/min) | .31, .01 | 4.85 |
| Low-frequency Word Reading (items/min) | .29, .01 | 3.19 |
| Pseudoword Reading (items/min) | .27, .02 | 2.09 |


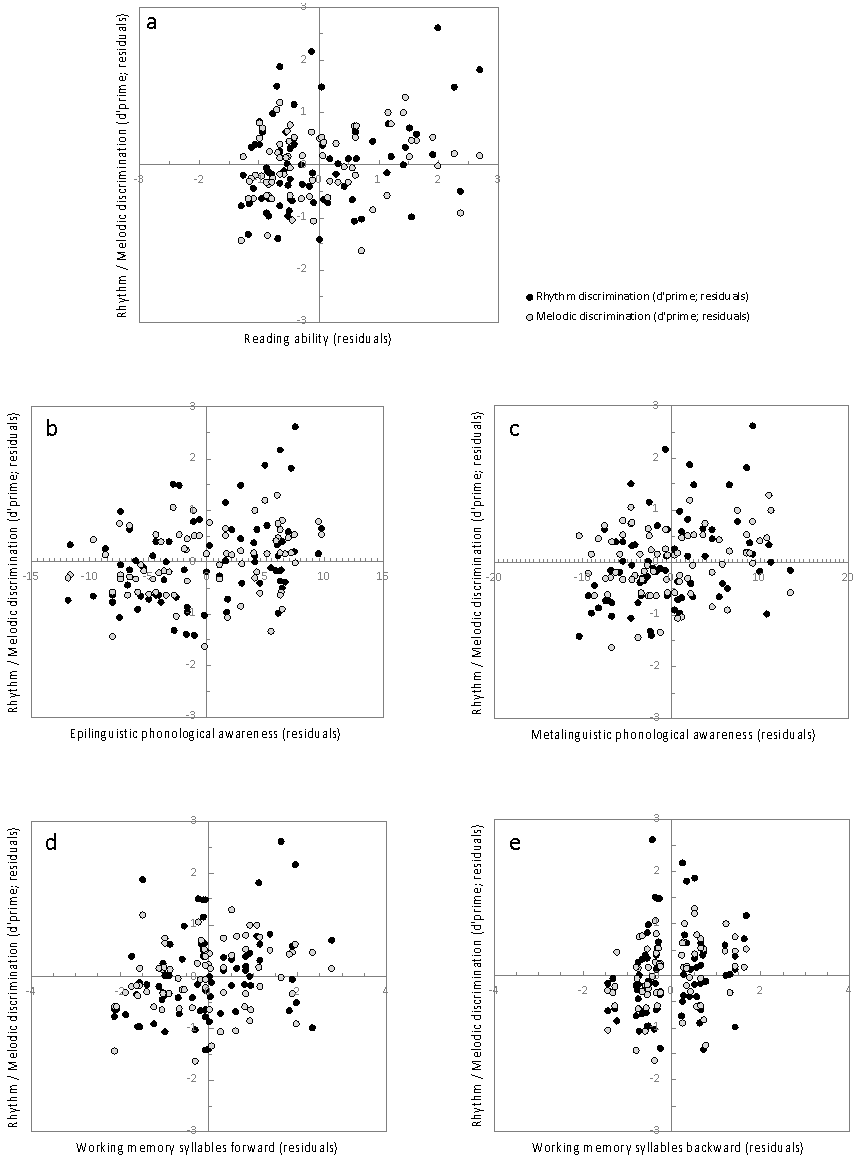


**Supplementary Figure S1.** Scatterplots showing the relationship between rhythm and melodic discrimination (*d’* scores) and (a) reading ability, (b) epilinguistic phonological awareness, (c) metalinguistic phonological awareness, (d) working memory of syllables forward and (e) backward, after removing the effects of cognitive ability.


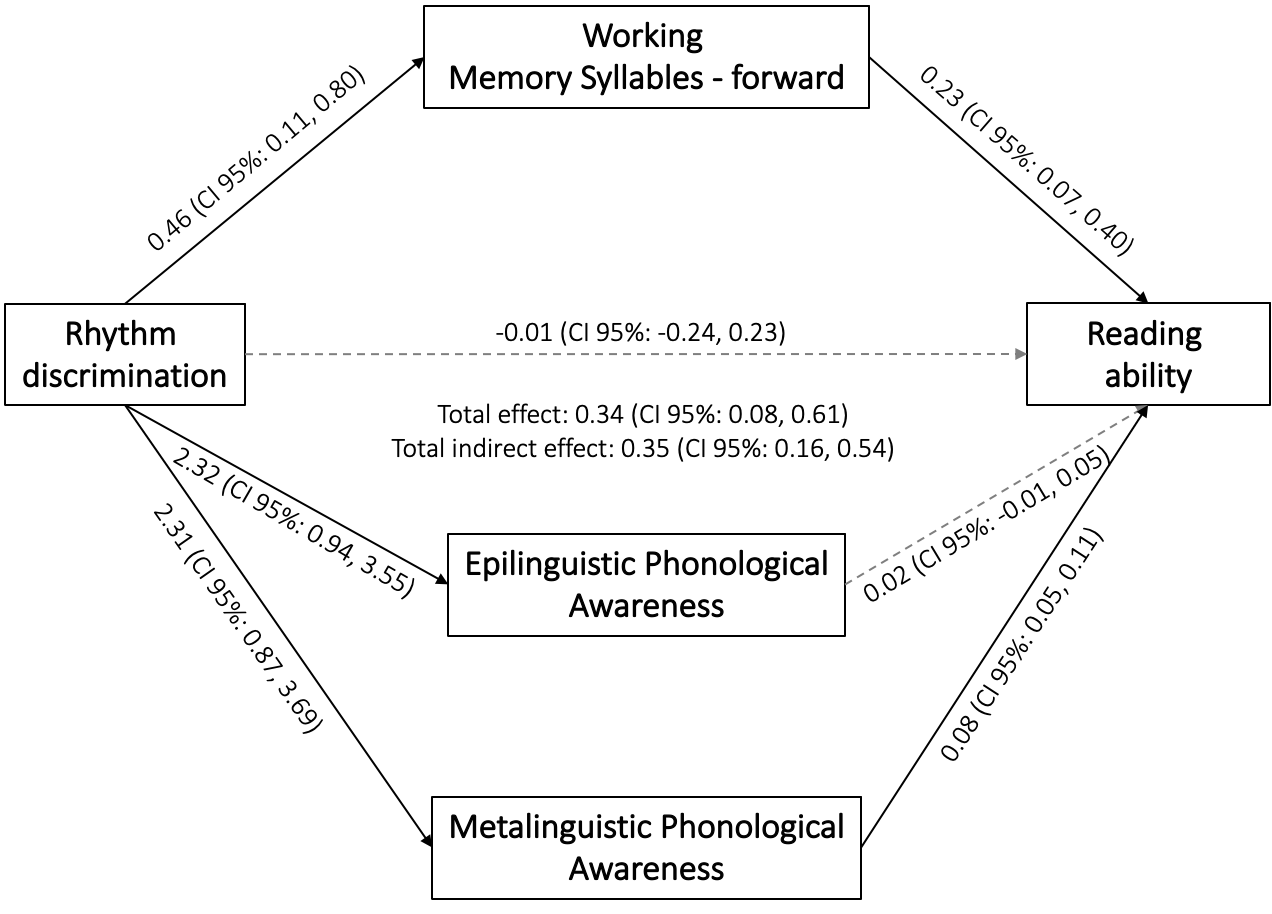


**Supplementary Figure S2.** Parallel mediation model (*N* = 73) depicting the mediation effect of working memory of syllables forward, epilinguistic phonological awareness, and metalinguistic phonological awareness on the association between rhythm discrimination and reading ability. Effects were considered significant when the CIs did not include 0. Inference was based on percentile bootstrap 95% confidence intervals (CIs) with 20,000 samples.


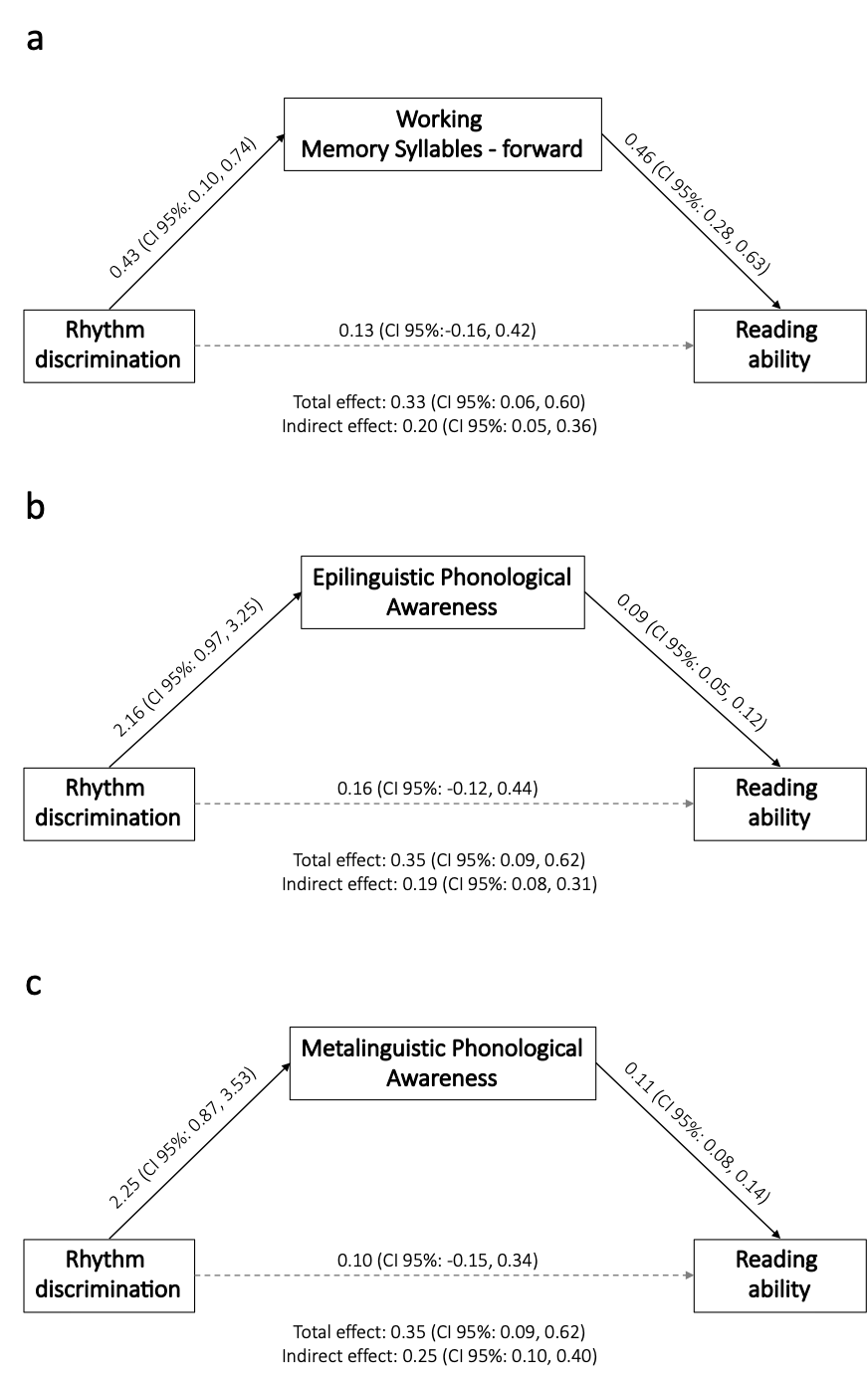


**Supplementary Figure S3.** Mediation models depicting the mediation effect of working memory of syllables forward (a), epilinguistic phonological awareness (b), and metalinguistic phonological awareness (c) on the association between rhythm discrimination and reading ability. The models were controlled for cognitive ability. Effects were considered significant when the CIs did not include 0. Inference was based on percentile bootstrap 95% confidence intervals (CIs) with 20,000 samples.
